# Arbeitsplatzbezogenes Belastungserleben und psychische Gesundheit der Beschäftigten im Gesundheitswesen während der COVID-19-Pandemie: Risiko- und Schutzfaktoren aus der VOICE-Studie

**DOI:** 10.1007/s00103-024-03954-x

**Published:** 2024-09-27

**Authors:** Yesim Erim, Franziska Geiser, Petra Beschoner, Lucia Jerg-Bretzke, Kerstin Weidner, Christian Albus, Andreas M. Baranowski, Sabine Mogwitz, Eva Morawa

**Affiliations:** 1 Psychosomatische und Psychotherapeutische Abteilung, Universitätsklinikum Erlangen, Friedrich-Alexander-Universität Erlangen-Nürnberg (FAU), Schwabachanlage 6, 91054 Erlangen, Deutschland; 2 Klinik und Poliklinik für Psychosomatische Medizin und Psychotherapie, Universitätsklinik Bonn, Universität Bonn, Bonn, Deutschland; 3 Klinik für Psychosomatische Medizin und Psychotherapie, Universitätsklinik Ulm, Ulm, Deutschland; 4 Klinik für Psychosomatische Medizin und Psychotherapie, Klinik Christophsbad, Göppingen, Deutschland; 5 Klinik und Poliklinik für Psychotherapie und Psychosomatik, Universitätsklinikum C. G. Carus, Medizinische Fakultät, Technische Universität Dresden, Dresden, Deutschland; 6 Klinik und Poliklinik für Psychosomatik und Psychotherapie, Universitätsklinik und Medizinische Fakultät, Universität zu Köln, Köln, Deutschland

**Keywords:** COVID-19-Pandemie, Gesundheitspersonal, Kohärenzgefühl, Work-Family-Conflict, Effort-Reward-Imbalance, COVID-19 pandemic, Healthcare workers, Sense of Coherence, Work-Family Conflict, Effort-Reward Imbalance

## Abstract

**Hintergrund:**

Beschäftigte im Gesundheitswesen haben ein erhöhtes Risiko für Depressionen und Angstsymptome und insbesondere während der COVID-19-Pandemie war medizinisches Personal vielseitig gefordert. Ziel der VOICE-Studie war es, Risiko- und Schutzfaktoren für arbeitsplatzbezogenes Belastungserleben und psychische Gesundheit zu untersuchen.

**Methode:**

Im Verbund von 5 psychosomatischen Universitätskliniken (Erlangen, Ulm, Bonn, Köln und Dresden) wurde ab Frühjahr 2020 eine multizentrische, webbasierte und prospektive Befragung (VOICE-Studie) initiiert. An der Studie nahmen zu 5 Messzeitpunkten mehr als 25.000 Personen teil.

**Ergebnisse:**

Von 3678 während der ersten Pandemiewelle untersuchten Mitarbeitenden im Krankenhaussetting waren 17,4 % bzw. 17,8 % der Ärztinnen und Ärzte, 21,6 % bzw. 19,0 % der Pflegekräfte und 23,0 % bzw. 20,1 % der medizinisch-technischen Assistenteninnen und Assistenten (MTA) von Depressions- und Angstsymptomen in klinisch relevantem Ausmaß betroffen. Die wichtigsten Risikofaktoren für eine depressive bzw. Angstsymptomatik waren unzureichende Erholung in der Freizeit, erhöhter Alkoholkonsum, geringeres Vertrauen in die Kolleginnen und Kollegen in schwierigen Arbeitssituationen sowie erhöhte Angst, sich mit COVID-19 zu infizieren. Prädiktoren für eine erhöhte posttraumatische Symptomatik waren erhöhte generalisierte Angst und Depressivität sowie erhöhte Angst vor Ansteckung der Angehörigen. Als protektive Faktoren fungierten Kohärenzgefühl, soziale Unterstützung, Optimismus und Belohnungsniveau.

**Diskussion:**

Die psychischen Auswirkungen arbeitsplatzbezogener Belastung in der Pandemie stellten sich als bedeutsam dar. Daher sind regelmäßige Screening- und Präventionsprogramme zur psychischen Gesundheit für Beschäftigte im Gesundheitswesen angezeigt.

## Abstract (en)

**Background:**

Healthcare workers have an increased risk of depression and anxiety, and medical staff have faced a wide variety of challenges, especially during the COVID-19-pandemic. The aim of the VOICE study was to investigate risk and protective factors for workplace-related stress experience and mental health.

**Method:**

A multicentre, web-based and prospective survey (VOICE study) was initiated in the spring of 2020 by a network of five psychosomatic university clinics (Erlangen, Ulm, Bonn, Cologne and Dresden). More than 25,000 respondents took part in the study at five measurement points.

**Results:**

Of 3678 employees examined in a hospital setting during the first wave of the pandemic, 17.4% and 17.8% of physicians, 21.6% and 19.0% of nurses and 23.0% and 20.1% of medical-technical assistants (MTA) were affected by symptoms of depression and anxiety, respectively, to a clinically relevant extent. The most important risk factors for depressive and anxiety symptoms were insufficient relaxation during leisure time, increased alcohol consumption, lower trust in colleagues in difficult work situations and increased fear of becoming ill with COVID-19. Predictors for increased post-traumatic symptoms were increased generalized anxiety and depression as well as increased fear of infecting family members. Sense of coherence, social support, optimism and reward level acted as protective factors.

**Discussion:**

The psychological effects of workplace-related stress during the pandemic were found to be significant. Therefore, regular mental health screening and prevention programmes for healthcare workers are indicated.

## Hintergrund

Weltweit und in Deutschland weist die Studienlage seit Langem auf ein hohes Risiko für klinisch auffällige Werte bzgl. depressiver und Angstsymptome bei Beschäftigten im Gesundheitswesen hin [1, 2]. Während der COVID-19-Pandemie in den Jahren 2020 bis 2023 war insbesondere das medizinische Personal vielseitig gefordert. Mit dem Ziel, Informationen zu Risiko- und protektiven Faktoren prospektiv durch Online-Umfragen zu sammeln und eine Datenbasis für Optimierungsvorschläge zu entwickeln, etablierte eine Arbeitsgruppe von 5 psychosomatischen Universitätsabteilungen die vorliegende longitudinale Online-Untersuchung. Da die Daten durch Selbstangaben der Mitarbeitenden entstanden und ihren Bedarfen eine Stimme geben sollten, wurde die Studie unter dem Titel VOICE geführt.

### In der VOICE-Studie untersuchte Risiko- und protektive Faktoren von medizinischem Personal während der COVID-19-Pandemie

Die Arbeitsgruppe analysierte in einer systematischen Übersichtsarbeit den Stand der Literatur zu den Auswirkungen der COVID-19-Pandemie auf die psychische Gesundheit des medizinischen Personals und den moderierenden Effekt von psychosozialen Ressourcen im ersten Pandemiejahr. Ausgewertet wurden dabei 46 Studien [3]. Untersuchte Variablen psychischer Belastung umfassten v. a. Stresserleben, Depressivität, Ängstlichkeit und posttraumatischen Stress. Als häufigste arbeitsplatzbezogene Belastungsfaktoren konnten erhöhtes Infektionsrisiko, Infektionsangst, gestiegene Arbeitsanforderungen bzw. Arbeitsüberlastung, unzureichende fachliche Vorbereitung und mangelnde wahrgenommene Unterstützung durch die Institution identifiziert werden. Sowohl die vorgenannten Variablen als auch die nachfolgenden pandemiespezifischen Belastungs- und Schutzfaktoren wurden in den Datensatz der VOICE-Studie aufgenommen.

Die *Vereinbarkeit von Beruf und Familie* ist eine Problematik, die alle Berufsgruppen betrifft, jedoch sind Gruppen mit Schichtarbeit, unregelmäßigen Arbeitszeiten und hoher Arbeitsbelastung, wie die Beschäftigten im Gesundheitswesen, häufiger betroffen [4]. Die Unvereinbarkeit von Anforderungen des Arbeitsplatzes und den familiären Pflichten als sozialer Rollenkonflikt umfasst sowohl den problematischen Einfluss des Berufes auf die Familie (Work-Family-Conflict, WFC) als auch den der Familie auf das Arbeitsleben (Family-Work-Conflict, FWC; [5]). Während der COVID-19-Pandemie ist diese Thematik vermehrt in den Blick gerückt, da die Kita- und Schulschließungen die Doppelbelastung verstärkt haben dürften [6–8].

Das *Effort-Reward-Imbalance-(ERI-) Modell* von Siegrist [9] geht davon aus, dass eine Balance zwischen Leistung (Effort) und Belohnung (Reward) einen wichtigen Beitrag zum Erhalt der Gesundheit arbeitender Menschen leistet, während die Verletzung dieser Balance das Risiko stressassoziierter Erkrankungen erhöht. In der VOICE-Studie wurde der psychosoziale Arbeitsstress nach diesem Modell gemessen.

*Moral Distress (MD)* bezeichnet die psychische Belastung, die entsteht, wenn eine Person eine bestimmte Handlung als moralisch richtig ansieht, aber aufgrund institutioneller Beschränkungen daran gehindert wird, gemäß ihren moralischen Überzeugungen zu handeln [10]. Während der COVID-19-Pandemie sah sich das Gesundheitspersonal insbesondere in ambulanten Einrichtungen wie sozialpädiatrischen Zentren, die durch die Kontaktbeschränkungen daran gehindert waren, Hilfestellungen zu geben, mit Abwägungsprozessen zwischen individuellem Patientenwohl und öffentlichem Gesundheitsschutz sowie mit der Beeinträchtigung der Patientenversorgung durch Hygienemaßnahmen, fehlende Schutzausrüstung und Personalknappheit konfrontiert [11].

*Psychische Resilienz* bezeichnet die Fähigkeit eines Individuums, effektiv mit Stress, Herausforderungen, Traumata oder widrigen Lebensumständen umzugehen und sich von diesen zu erholen [12]. Eine höhere Resilienz ist mit einer besseren psychischen Gesundheit sowohl in der Allgemeinbevölkerung [13] als auch bei Beschäftigten des Gesundheitswesens [14] assoziiert. Dies gilt auch für soziale Unterstützung [15]. Aus diesem Grund wurden auch diese Faktoren in der VOICE-Studie gemessen, wobei zu betonen ist, dass die Resilienz an sich nicht erfasst werden kann, sondern die subjektive Resilienzzuschreibung. Im Rahmen der VOICE-Studie wurde ein mit der Resilienz verwandtes Konstrukt untersucht, nämlich das Kohärenzgefühl [16], das als eine globale Lebenseinstellung betrachtet werden kann, welche das Leben als verstehbar, handhabbar und sinnvoll erscheinen lässt.

### Fragestellungen

Welche Ausmaße hatten psychische Belastungen wie Depressivität, generalisierte Angst und posttraumatische Symptomatik zu Beginn der Pandemie und wie entwickelten sie sich im Verlauf? Welcher Zusammenhang besteht zwischen den psychischen Belastungen und diversen Risikofaktoren wie der pandemiespezifischen Belastung am Arbeitsplatz, moralischen Konflikten und der Vereinbarkeit von Beruf und Familie und Schutzfaktoren wie Kohärenzgefühl, soziale Unterstützung und Optimismus?

## Methode

Die VOICE-Studie ist eine multizentrische, webbasierte und prospektive Befragung der psychosomatischen Universitätskliniken Erlangen, Bonn, Ulm, Dresden und Köln. Dieses Verbundprojekt wurde als Teil des Projektes „egePan Unimed“ im Rahmen des Nationalen Netzwerks Universitätsmedizin (NUM) während der zweiten Erhebungswelle vom Bundesministerium für Bildung und Forschung (BMBF) gefördert. Die Rekrutierung von Teilnehmenden erfolgte über E‑Mail-Listen oder Online-Mitarbeiterportale der psychosomatischen Universitätskliniken. Des Weiteren wurden auch nichtuniversitäre Krankenhäuser/Kliniken und verschiedene medizinische Fachgesellschaften und Online-Plattformen gebeten, für die Teilnahme an der Befragung zu werben.

Einschlusskriterien waren ein Mindestalter von 18 Jahren, Arbeit im Gesundheitswesen, Wohn‑/Arbeitsort in Deutschland und ausreichende Deutschkenntnisse. Die ca. 15- bis 20-minütige Befragung bestand zum ersten Messzeitpunkt (20.04.–05.07.2020) aus ca. 77 Items. Diese Zahl variierte leicht zu späteren Zeitpunkten, da ein Teil der Fragebogenbatterie an die jeweilige Pandemiesituation angepasst wurde, um bestimmte relevante Variablen neben dem konstanten Hauptanteil erfassen zu können. Dieser umfasste folgende Konstrukte: Depressivität, Angst, posttraumatische Belastungsstörung (PTBS), ERI, WFC und FWC, MD, Kohärenzgefühl, soziale Unterstützung, Optimismus und Lebensqualität (LQ). Außerdem wurden Arbeitsbedingungen, COVID-19-bezogene Probleme, soziodemografische und arbeitsplatzbezogene Merkmale erfragt.

Die Auswertungen fokussierten neben spezifischen Fragestellungen auch spezifische Professionen oder Zeiträume, sodass sich im Folgenden unterschiedliche Anzahlen (*N*) aus den Teilstudien ergeben.

## Ergebnisse

Zwischen April 2020 und Juni 2023 haben an mindestens einem der 5 stattgefundenen Messzeitpunkte (T1: 20.04.–05.07.2020, *N* = 8071; T2: 17.11.2020–07.01.2021, *N* = 7202; T3: 28.05.–16.07.2021, *N* = 3463; T4: 07.02.–01.05.2022, *N* = 4536 und T5: 17.04.–04.06.2023, *N* = 2816) insgesamt mehr als 25.000 Beschäftigte im Gesundheitswesen teilgenommen.

### Prävalenz und Prädiktoren klinisch relevanter Depressions- und Angstsymptome zu Beginn der COVID-19-Pandemie

Die erste Befragung [17] fokussierte die Arbeitsbedingungen sowie potenzielle Risiko- und Schutzfaktoren für depressive und Angstsymptome (= Outcomes der ungünstig wirkenden Belastungsfaktoren) während der COVID-19-Pandemie bei 3678 Beschäftigten im Krankenhaus-Setting (Ärztinnen/Ärzte, Pflegekräfte und medizinisch-technische Assistenteninnen und Assistenten (MTA)) im Rahmen der ersten Erhebungswelle von April bis Juli 2020. Die Prävalenz klinisch relevanter Depressions- und Angstsymptome betrug 17,4 % bzw. 17,8 % bei Ärztinnen und Ärzten, 21,6 % bzw. 19,0 % bei Pflegekräften und 23,0 % bzw. 20,1 % bei MTA. Alle 3 Berufsgruppen wiesen im Vergleich zu einem repräsentativen Sample aus der deutschen Allgemeinbevölkerung vor der Pandemie [18] signifikant erhöhte Werte im Patient-Health-Questionnaire‑2 (PHQ-2) und in der Generalized-Anxiety-Disorder-Scale‑2 (GAD-2) auf, jedoch niedrigere Scores im Vergleich zu einer großen, aber nicht repräsentativen Stichprobe aus der Allgemeinbevölkerung in der frühen Phase der Pandemie [19]. Höhere Werte für depressive Symptome waren mit unzureichender Erholung in der Freizeit, erhöhtem Alkoholkonsum und geringerem Vertrauen in Kolleginnen und Kollegen in schwierigen Arbeitssituationen assoziiert. Darüber hinaus standen erhöhte Angstwerte im Zusammenhang mit der Angst, sich mit COVID-19 zu infizieren.

### Entwicklung der psychischen Belastung während der Pandemie bei Ärztinnen und Ärzten und Pflegekräften

Zu vier Messzeitpunkten zwischen April 2020 und Mai 2022 wurden Depressions- und Angstsymptome (= Outcome-Variablen) bei Ärztinnen und Ärzten in deutschen Krankenhäusern (*N* = 340) untersucht [20]. Sie zeigten einen signifikanten Anstieg der depressiven Symptome von T1 (April bis Juli 2020) zu T4 (Februar bis Mai 2022) und der Angstsymptome von T1 zu T2 (November 2020 bis Januar 2021). Frauen waren signifikant ängstlicher als Männer. 18- bis 40-Jährige wiesen höhere Depressivitätswerte auf als die über 50-Jährigen. Im Vergleich zur Allgemeinbevölkerung [21] berichteten die Ärztinnen und Ärzte signifikant erhöhte PHQ-2- und GAD-2-Scores (über alle Messzeitpunkte hinweg).

Den Fokus einer weiteren longitudinalen Analyse [22] bildete der Verlauf der Depressions- und Angstsymptome bei Pflegekräften (*N* = 507). Im Vergleich mit T1 wurde zu allen weiteren Messzeitpunkten eine signifikante Zunahme der beiden Outcomes beobachtet. Ferner zeigten die Pflegekräfte zu allen vier Messzeitpunkten signifikant höher ausgeprägte Depressions- und Angstsymptome als die deutsche Allgemeinbevölkerung.

### Zeichen von traumatischer Belastung

Während der COVID-19-Pandemie waren insbesondere Beschäftigte im Gesundheitswesen nicht nur akuten und chronischen Stressoren, sondern auch unvorhersehbaren potenziell traumatischen Ereignissen ausgesetzt. Als mögliche psychische Folge dieser Erfahrungen können sich Symptome einer PTBS einstellen [23]. Während der ersten Pandemiewelle wurden bei 4724 Beschäftigten im Gesundheitswesen [24] als relevante statistische Prädiktoren für eine erhöhte PTBS-Symptomatik (= Outcome-Variable) eine erhöhte generalisierte Angst und Depressivität, erhöhte Angst vor Ansteckung von Angehörigen sowie die Profession innerhalb des Gesundheitswesens (MTA mit erhöhter Symptomatik im Vergleich zu Ärztinnen und Ärzten) identifiziert.

### MD

Pflegekräfte und MTA erlebten während der ersten Pandemiewelle eine höhere moralische Belastung (= Outcome-Variable) als Ärztinnen und Ärzte, Psychologinnen und Psychologen, Psychotherapeutinnen und -therapeuten sowie Seelsorgerinnen und Seelsorger. Diese Belastung korrelierte signifikant sowohl mit einer hohen Belegungsrate auf Station und Kontakt mit SARS-CoV-2-Infizierten als auch mit berichteten depressiven und Angstsymptomen. Insbesondere Pflegekräfte zeigten in allen Arbeitsbereichen ein Niveau moralischer Belastung, das vor der Pandemie hauptsächlich im intensiv- und notfallmedizinischen Bereich beobachtet wurde [25].

### Moderatoren und psychische Ressourcen in der Pandemie

Während der COVID-19-Pandemie zeigte sich für Beschäftigte im Gesundheitswesen im Vergleich zu Werten in der Allgemeinbevölkerung vor der Pandemie ein hohes Maß an psychischer Resilienz [26], das mit einem höheren Alter assoziiert war. Sowohl ein geringes individuelles Kohärenzgefühl als auch eine hohe pandemiebedingte Stressbelastung trugen unabhängig voneinander zu Angst- und Depressionssymptomen (= Outcome-Variablen, auch in den anderen Studien unter Abschn. „Moderatoren und psychische Ressourcen in der Pandemie“) bei. Zusätzlich moderierte das Kohärenzgefühl die Beziehung zwischen Stressbelastung und Angstsymptomen [27].

Ein stärkeres Kohärenzgefühl [16] hing ebenfalls mit weniger Angst- und Depressionssymptomen zusammen. Auch soziale Unterstützung zeigte eine positive Assoziation mit der psychischen Gesundheit, jedoch mit geringerer Effektstärke [28]. Religiosität, allerdings gemessen über nur ein Item, wies demgegenüber kaum einen Zusammenhang mit Angst oder Depression auf.

Ein signifikanter negativer Zusammenhang zwischen sozialer Unterstützung und Optimismus einerseits und depressiven und Angstsymptomen andererseits wurde konstatiert, wobei soziale Unterstützung und Optimismus mit den beiden Indikatoren der psychischen Belastung stärker assoziiert waren als soziodemografische oder pandemiebezogene Faktoren [29].

Zu unterstreichen ist ferner die Bedeutung der Teamkohärenz, die als ein „Sich-Verlassen-Können auf die Kolleginnen und Kollegen, wenn es bei der Arbeit schwierig wird“, operationalisiert worden war und sich in der VOICE-Studie in mehreren Analysen, in verschiedenen Berufsgruppen und Settings (z. B. [17, 30]) als signifikanter und relevanter Schutzfaktor der psychischen Gesundheit für Mitarbeitende im Gesundheitswesen erwiesen hat.

### ERI

Wir untersuchten 6174 Beschäftigte im Gesundheitswesen zu beruflichem Stress nach dem ERI-Modell von Siegrist [9]. Die ERI (ERI-Ratio >1; = Outcome-Variable) wurde bei der Hälfte der Stichprobe (50,9 %) und signifikant häufiger bei Frauen (51,8 %) als bei Männern (47,8 %) detektiert [31]. Am häufigsten wurde sie bei Pflegekräften (62,8 %) und MTA (58,8 %), am seltensten bei Psychologinnen und Psychologen (37,8 %) und Ärztinnen und Ärzten (41,8 %) festgestellt.

Hinsichtlich des Ausmaßes von „Effort“ waren keine signifikanten geschlechtsspezifischen Differenzen zu beobachten, beim „Reward“ erzielten Männer aber signifikant höhere Werte als Frauen. Pflegekräfte erreichten die höchsten Werte für die Skala „Effort“, Psychologinnen und Psychologen hingegen die geringsten Werte. MTA und Pflegekräfte erreichten die niedrigsten Werte für „Reward“ im Vergleich zu allen anderen Berufsgruppen, Ärztinnen und Ärzte sowie Psychologinnen und Psychologen die höchsten.

### WFC

Die Analyse der Skalen WFC und FWC (= Outcome-Variablen) zeigte eine Zunahme der Konflikte zwischen Arbeit und Familie im Verlauf der Pandemie. Insbesondere nahm der WFC von T1 auf T2 zu, um dann zu T3 leicht abzufallen und zu T4 wieder anzusteigen. Ähnlich verlief die Entwicklung des FWC. Frauen empfanden signifikant weniger Stress. Die 31- bis 50-Jährigen waren signifikant stärker belastet als die 18- bis 30-Jährigen, während die über 60-Jährigen am wenigsten vom WFC und FWC betroffen waren.

### Besondere Gruppen und ihre mentale Belastung während der Pandemie

Besonderes Interesse der VOICE-Studie galt nicht nur der Identifizierung von Risikofaktoren der psychischen Gesundheit des medizinischen Personals, sondern ebenfalls der Aufdeckung vulnerabler Subgruppen mit ihren je spezifischen Belastungen und Bedarfen.

#### Sozialpädiatrische Zentren

An der VOICE-Studie nahm eine große Zahl von Mitarbeitenden in sozialpädiatrischen Zentren (z. B. (Neuro‑)Pädiaterinnen und -pädiater, Psychologinnen und Psychologen, Logopädinnen und Logopäden) teil (*N* = 1291; [32]). Die besondere Belastung war, dass sozialpädiatrische Zentren zeitweise geschlossen wurden und auch Hausbesuche nicht möglich waren, was zu moralischen Konflikten führte. Die Häufigkeit klinisch relevanter Ausprägung depressiver und generalisierter Angstsymptome (= Outcome-Variablen) fiel ähnlich wie beim medizinischen Personal aus. Die Studienergebnisse zeigen, dass auch Mitarbeitende, die nicht direkt an der Versorgung von akut Erkrankten beteiligt waren, erhebliche Belastungen aufwiesen, die sich zum Teil nicht von denen der direkt betroffenen Fachkräfte unterschieden.

#### Niedergelassene Ärztinnen und Ärzte

Bei 848 niedergelassenen Ärztinnen und Ärzten wurde der Verlauf der psychischen Belastung sowie die LQ (= Outcome-Variablen) im Vergleich mit 458 Krankenhausärztinnen und -ärzten analysiert [30]. Zu T1 zeigte sich bei Ersteren eine höhere Depressivität bei mit Krankenhausärztinnen und -ärzten vergleichbarer subjektiver LQ und aktuell empfundener Belastung. Zu T2 berichteten die niedergelassenen Ärztinnen und Ärzte höhere Werte aktueller Belastung, Depressivität und Ängstlichkeit, WFC und weniger LQ verglichen mit der anderen Gruppe. Nahezu alle erhobenen Belastungsparameter stiegen von T1 zu T2 innerhalb der Kohorten der Niedergelassenen an. Risikofaktoren für die psychische Belastung waren hier der WFC, Besorgnis um die Sicherheit der Patienteninnen und Patienten, Angst, Patientinnen und Patienten priorisieren zu müssen, und die Belastung durch Restriktionen von Sozialkontakten in der Freizeit, während der empfundene Schutz durch örtliche Autoritäten, das Vertrauen in Kolleginnen und Kollegen in schwierigen Situationen und allgemeine soziale Unterstützung Schutzfaktoren darstellten.

#### Beschäftigte mit Migrationshintergrund

Bei 7187 einheimisch deutschen Beschäftigten sowie solchen mit Migrationshintergrund (MH; Anteil = 10,9 %) im Gesundheitssektor wurden zum ersten Messzeitpunkt die depressiven und generalisierten Angstsymptome (= Outcome-Variablen) untersucht, wobei verschiedene Migrantensubgruppen (z. B. erste vs. zweite Migrantengeneration; Herkunft aus Ländern mit niedrigem/mittlerem vs. hohem Einkommen) analysiert wurden [33]. Personen mit MH aus Ländern mit niedrigem/mittlerem Einkommen zeigten häufiger klinisch relevante depressive Symptome (PHQ-2 ≥ 3) als solche aus Ländern mit hohem Einkommen; bzgl. klinisch relevanter Angstwerte (GAD-2 ≥ 3) wurden keine Unterschiede festgestellt. Nach Kontrolle relevanter Variablen unterschieden sich die einheimischen Mitarbeiterinnen und Migranten nicht von den einzelnen Migrantengruppen in Bezug auf den Schweregrad von depressiver und Angstsymptomatik.

### Reaktionen auf die Pandemiebedingungen

Eine zentrale Rolle in der Pandemiebewältigung kam der *COVID-19-Impfung* zu. Da das Gesundheitspersonal eine Schlüsselrolle bei der Bekämpfung der Pandemie innehatte, war seine Impfbereitschaft von großer Relevanz. Diese wurde im Rahmen der zweiten Erhebungswelle zwischen November 2020 und Januar 2021, also vor der generellen Einführung der Impfstoffe, bei 6217 Beschäftigten im deutschen Gesundheitssektor untersucht [34]. Die Impfbereitschaft (= Outcome-Variable) der Gesamtstichprobe war mit 65,3 % insgesamt mäßig, wobei von allen befragten Berufsgruppen Ärztinnen und Ärzte die höchste Bereitschaft aufwiesen. Folgende Merkmale waren mit einer höheren Impfbereitschaft assoziiert: männliches Geschlecht, Alter >40 Jahre, keine Kinder, kein MH, keine Tätigkeit in der direkten Patientenversorgung, Zugehörigkeit zu einer COVID-19-Risikogruppe, Zugehörigkeit zur Berufsgruppe der Ärztinnen und Ärzte sowie der Psychologinnen und Psychologen im Vergleich mit den Pflegekräften, ausreichende Informiertheit über COVID-19 und wahrgenommener Schutz durch Maßnahmen nationaler/lokaler Behörden und des Arbeitgebers, Angst vor Infektion sowie fehlende klinisch relevante depressive Symptomatik.

*Krankheitsbedingte Fehlzeiten* und der *Kündigungswunsch* von Gesundheits- und Krankenpflegerinnen und -pflegern verschärften den bereits bestehenden Mangel an Pflegekräften während der COVID-19-Pandemie in Deutschland und anderen Ländern [35]. Im Rahmen der dritten Erhebungswelle analysierten wir aus diesem Grund die Häufigkeit und die mit Arbeitsunfähigkeit (AU) und Kündigungsabsichten (= Outcome-Variablen) assoziierten Faktoren bei Pflegekräften (*N* = 757). Die Absicht, das Arbeitsverhältnis zu beenden, bestand bei 18,9 %. Niedrigere Belohnungsniveaus (ERI-Reward-Skala), ein Wechsel der Arbeitsabteilung (Versetzung) während der Pandemie, Teilzeitarbeit und höhere Depressionswerte stellten signifikante Prädiktoren für den Kündigungswunsch dar. Ein höheres wahrgenommenes Belohnungsniveau schien sowohl die AU-Tage als auch die Kündigungsabsicht zu verringern.

## Diskussion

Die vorliegende Arbeit gibt einen Einblick in die wichtigsten Ergebnisse der VOICE-Studie, die so vielfältig sind, dass im Folgenden nur ausgewählte zentrale Ergebnisse diskutiert werden. In diesem Verbundprojekt konnte eine gute Forschungsinfrastruktur etabliert werden, die es ermöglicht hat, innerhalb kurzer Zeiträume eine große Anzahl Mitarbeitender im Gesundheitsbereich zu vielen Aspekten zeitökonomisch zu befragen. Regelmäßiger engagierter und kooperativer Austausch innerhalb der Arbeitsgruppe hat dazu beigetragen, dass die VOICE-Studie nun schon seit über 4 Jahren fortbesteht und insgesamt 20 Publikationen in internationalen Fachjournalen vorweisen kann. Sehr wünschenswert wäre eine Verstetigung dieses gelungenen Verbundprojektes, z. B. in Form einer Panel-Befragung des medizinischen Personals.

### Ausmaß und Verlauf der psychischen Belastung während der Pandemie

Die höchste psychische Belastung in Form von depressiven und Angstsymptomen (= Outcomes der ungünstig wirkenden Belastungsfaktoren) zu T1 wiesen die MTA, gefolgt von Pflegekräften und Ärztinnen und Ärzten auf. Die Belastung nahm während der Pandemie zu und war zu jedem Zeitpunkt signifikant höher als die Werte der Allgemeinbevölkerung. Eine positive Assoziation wurde zwischen psychischer Belastung und infektionsbezogener Angst sowie mit unzureichender Erholung und Alkoholkonsum festgestellt. Möglicherweise wird Alkohol zur Bekämpfung der Erschöpfung eingesetzt; umgekehrt könnte ein höherer Alkoholkonsum aber auch mit einer von vornherein höheren Vulnerabilität zusammenhängen. Depressions- und Suchtprävention scheinen besonders in Krisenzeiten wie einer Pandemie eine hohe Bedeutung zu erlangen. Zu allen Messzeitpunkten wurde eine negative Korrelation mit einer guten Teamkohäsion festgestellt. Teamorientierte präventive oder interventionelle Maßnahmen könnten demnach die psychische Gesundheit von Teams unter Belastung stärken. Inspiriert durch diese Ergebnisse hat das Erlanger Team den Erlanger Fragebogen zur Teamkohärenz am Arbeitsplatz entwickelt und validiert [36], der in der sechsten Erhebungswelle der VOICE-Studie eingesetzt werden wird.

### ERI

Bezüglich des psychosozialen Arbeitsstresses während der Pandemie zeigte sich, dass mehr als die Hälfte der Befragten unter hohem Arbeitsstress im Sinne des ERI-Modells leidet. Dabei sind Frauen signifikant häufiger hoch belastet als ihre männlichen Kollegen, was auf die signifikant niedrigeren Werte in der Reward-Skala der Frauen zurückzuführen ist. Für die Effort-Skala wurden dagegen keine geschlechtsspezifischen Effekte gefunden. Frauen und Männer bewerten ihre Arbeitsanstrengung ähnlich, Männer fühlen sich aber signifikant stärker sozial und monetär belohnt. Dieses Ergebnis ist auch deshalb hervorzuheben, weil etwa 77 % der Befragten weiblich waren. Eine Erklärung für diesen geschlechtsspezifischen Unterschied könnte sein, dass die schwierige Vereinbarkeit von Beruf und Familie im medizinischen Berufsfeld und die fehlende Zeit für die Care-Arbeit in erster Linie Frauen betrifft.

Im Berufsgruppenvergleich wiesen die Pflegekräfte mit zwei Dritteln die höchste Prävalenz einer ERI auf, gefolgt von der Gruppe der MTA. Erheblich niedrigere Anteile hoch Belasteter zeigen die psychologischen und ärztlichen Berufsgruppen. Alle medizinisch Beschäftigten haben in der Regel eine hohe Arbeitsbelastung, die jedoch in den Berufsfeldern der Pflege und MTA mit geringerer sozialer und monetärer Entlohnung verbunden ist.

### WFC/FWC

Die Studie belegt deutlich, dass die Belastung durch eine konflikthafte Vereinbarkeit von Beruf und Familie im Verlauf der Pandemie signifikant zunahm. Vor allem konkrete verhältnispräventive Maßnahmen, wie z. B. die Möglichkeit der Unterbrechung der Arbeit im familiären Notfall [4], verlässliche Teilzeitregelungen und für alle Beschäftigten die Möglichkeit familienbedingter Auszeiten (Eltern- und Pflegezeiten), würden Verbesserungsmöglichkeiten bieten. Familienfreundliche Arbeitsbedingungen zeigen sich ebenfalls in einer Anpassung der Öffnungszeiten der Kindertagesstätten an die gegebenen Bedarfe (Arbeitszeiten) der Beschäftigten. Ein besonderes Augenmerk sollte dabei auf die besonders betroffene Altersgruppe der 31- bis 50-Jährigen gelegt werden.

### Protektive Faktoren

Erwartungsgemäß fanden wir Zusammenhänge zwischen protektiven Konstrukten wie Kohärenzgefühl, Optimismus und sozialer Unterstützung mit der psychischen Belastung. Der gefundene Moderationseffekt des Kohärenzgefühls auf die Beziehung zwischen Stressbelastung und psychischer Gesundheit bestätigt die Bedeutung des Resilienzkonzeptes. Die COVID-19-Pandemie hat mit neuen Herausforderungen nicht nur zu einer erhöhten generellen Stressbelastung geführt, sondern insbesondere neue moralische Dilemmata erzeugt, z. B. hinsichtlich der Bedürfnisse zwischen guter Versorgung und Arbeitsüberlastung oder bei der Priorisierung von akut knappen Ressourcen. Dass Pflegepersonal und MTA häufiger MD angaben, mag damit zusammenhängen, dass ihr Gestaltungsspielraum kleiner ist und dass sie stärker durch die Zunahme von Aufgaben in Laboren oder im Infektionsschutz belastet waren.

Es ist wichtig zu betonen, dass die medizinische Tätigkeit nicht nur als Belastung, sondern auch als Ressource angesehen werden kann. So zeigte das untersuchte medizinische Personal in der frühen Pandemiephase niedrigere depressive und Angst-Scores im Vergleich zu der Allgemeinbevölkerung [19]. Offenbar konnte das medizinische Personal die pandemiebezogene Belastung zumindest zu Beginn der Pandemie recht gut bewältigen, was u. a. an der Möglichkeit liegen könnte, agieren zu können und nicht passiv abwarten zu müssen.

Grundsätzlich ist es methodisch schwierig, aus querschnittlich erhobenen Korrelationen konkrete Empfehlungen abzuleiten. Die Tatsache, dass es Faktoren gibt, die sich statistisch als protektiv erwiesen haben, lässt nicht automatisch den Schluss zu, dass Programme zu deren Förderung Erfolg versprechend wären. So hat z. B. das als Eigenschaft gemessene Kohärenzgefühl auch genetische oder biografische Ursachen, die nicht beeinflussbar sind. Zudem interagiert das Kohärenzgefühl mit konkreten strukturellen Bedingungen, z. B. mit dem Vertrauen ins Team. Dieser Faktor war in unseren Studien mit geringerer Belastung verbunden und setzt beispielsweise voraus, dass die personellen und strukturellen Voraussetzungen für ein funktionierendes Team gegeben sind.

Zur Stärkung von Mitarbeitenden im Gesundheitswesen in einer Pandemiesituation gehört auch die Frage, wie ein Kohärenzgefühl (im Sinne der Wahrnehmung einer kritischen Situation als bewältigbar, verstehbar und sinnhaft) im Kontext heterogener medizinischer Settings unterstützt werden kann. Hierzu könnten Maßnahmen zur Verbesserung von Informationsvermittlung, Mitgestaltungsmöglichkeiten und Gelegenheiten zum Austausch über das eigene Erleben der Situation v. a. für Pflegekräfte und MTA im Rahmen von Inter- und Supervisionen gehören. Allerdings muss man kritisch anmerken, dass die Mitgestaltungsmöglichkeiten wie auch die Entscheidungsfreiheit zumindest in manchen Aspekten, wie z. B. der Impfpflicht, für das medizinische Personal stark eingeschränkt waren und dass „Impfgegner“ sehr unter Druck gesetzt wurden, sodass diese Thematik teilweise zu heftigen Diskussionen und Konflikten in Teams geführt haben dürfte. Für zukünftige Pandemien wäre es also von großer Relevanz, für solche konflikthaften Situationen konstruktive Lösungsvorschläge zu erarbeiten.

Bisher noch zu wenig beachtet sind Ausmaß und Ursachen für MD bei verschiedenen Berufsgruppen, sodass vertiefte Nachbefragungen notwendig sind.

Unterstützende Beziehungen im Privaten und am Arbeitsplatz wirkten sich positiv auf die psychische Gesundheit niedergelassener Ärztinnen und Ärzte aus. Das im Verlauf stabile, jedoch im Vergleich zu Klinikärztinnen und -ärzten geringere Vertrauen in Kolleginnen und Kollegen sowie der positive Einfluss des empfundenen Schutzes durch lokale Autoritäten waren hierbei wichtige Schutzfaktoren und sind bedeutend für zukünftige Krisen. Eine gezielte Prävention und Unterstützung für niedergelassene Ärztinnen und Ärzte durch Stärkung sozialer Ressourcen und stärkere Vernetzung wären denkbar.

## Fazit

Um die psychische Belastung und Erschöpfung bei Beschäftigten im Gesundheitswesen während Pandemien gering zu halten, sollten neben der Angst‑, Depressions- und Suchtprävention Maßnahmen darauf abzielen, günstiges gesundheitsrelevantes Verhalten (wie z. B. Impfverhalten) zu stärken, die Vereinbarkeit von Familie und Beruf zu berücksichtigen, die Balance zwischen Leistung und Anerkennung auch geschlechterspezifisch zu gewährleisten und pandemiebezogene Maßnahmen so zu kommunizieren, dass die Wahrnehmung einer kritischen Situation als verstehbar, bewältigbar und sinnhaft unterstützt werden kann. Spezielle Maßnahmen zur Entwicklung der Teamkohäsion erscheinen sehr relevant, um über die pandemische Situation hinaus die Zufriedenheit mit der Arbeit zu steigern.

Zukünftige Forschung sollte dieVOICE-Studie verstetigen, beispielsweise in Form einer Panel-Befragung des medizinischen Personals, und ferner die Methodik optimieren, z. B. eine prospektive Befragung einer konstanten Gruppe Beschäftigter im Gesundheitswesen u. a. unter Einsatz strukturierter klinischer Interviews durchführen, um nicht nur psychische Belastung, sondern auch psychische Erkrankungen wie Depression, Angststörung und PTBS sowie deren Schutz- und Risikofaktoren identifizieren zu können.
